# Comparison of complete renal response and mortality in early- and late-onset lupus nephritis: a multicenter retrospective study of a Japanese cohort

**DOI:** 10.1186/s13075-020-02271-3

**Published:** 2020-07-22

**Authors:** Kunihiro Ichinose, Mineaki Kitamura, Shuntaro Sato, Keita Fujikawa, Yoshiro Horai, Naoki Matsuoka, Masahiko Tsuboi, Fumiaki Nonaka, Toshimasa Shimizu, Remi Sumiyoshi, Tomohiro Koga, Shin-ya Kawashiri, Naoki Iwamoto, Takashi Igawa, Mami Tamai, Hideki Nakamura, Tomoki Origuchi, Tomoya Nishino, Atsushi Kawakami

**Affiliations:** 1 Department of Immunology and Rheumatology, Division of Advanced Preventive Medical Sciences, Nagasaki University Graduate School of Biomedical Sciences, 1-7-1 Sakamoto, Nagasaki, 852-8501 Japan; 2 Department of Nephrology, Nagasaki University Hospital, Nagasaki, Japan; 3 Clinical Research Center, Nagasaki University Hospital, Nagasaki, Japan; 4 Department of Rheumatology, JCHO Isahaya General Hospital, Isahaya, Japan; 5 Department of Rheumatology, Clinical Research Center, NHO Nagasaki Medical Center, Omura, Japan; 6 Nagasaki Medical Hospital of Rheumatology, Nagasaki, Japan; 7 Department of Internal Medicine, Sasebo City General Hospital, Sasebo, Japan; 8 Department of Rehabilitation Sciences, Nagasaki University Graduate School of Biomedical Sciences, Nagasaki, Japan

**Keywords:** Complete renal response, Lupus nephritis, Early onset, Late onset, Systemic lupus erythematosus

## Abstract

**Background:**

Most patients with systemic lupus erythematosus (SLE) progress to lupus nephritis (LN) within 5 years of their SLE diagnosis, although it is not uncommon for LN to develop at later time points. Here we evaluated the clinical features of early- and late-onset LN.

**Patients and methods:**

We retrospectively analyzed the cases of 184 of the 201 patients who underwent a renal biopsy at Nagasaki University Hospital and associated community hospitals between 1990 and 2016 and were diagnosed as having LN. Early onset was defined as the development of LN within the first 5 years after the patient’s SLE diagnosis, and late onset was defined as LN development > 5 years post-diagnosis. We analyzed the complete renal response (CR) at 6 and 12 months after induction therapy, the classification of renal pathology, and the mortality of the early- and late-onset LN groups.

**Results:**

The mean follow-up duration after the renal biopsy was 123 ± 85 months. There were 113 (61.4%) early-onset patients and 71 (38.6%) late-onset patients. A multivariate analysis revealed that the following factors were predictive of CR: at 6 months: female sex (odds ratio [OR] 3.93, 95% confidence interval [CI] 1.31–11.77, *p* = 0.010), proteinuria (OR 0.83, 95% CI 0.71–0.97, *p* = 0.009), index of activity (0–24) (OR 0.83, 95% CI 0.70–0.99, *p* = 0.030), and early-onset LN (OR 2.39, 95% CI 1.15–4.98, *p* = 0.018); at 12 months: female sex (OR 3.60, 95% CI 1.32–9.83, *p* = 0.013), mixed LN (OR 0.18, 95% CI 0.04–0.80, *p* = 0.024), index of activity (0–24) (OR 0.80, 95% CI 0.68–0.94, *p* = 0.007), and early-onset LN (OR 2.10, 95% CI 1.05–4.23, *p* = 0.035). In a Cox proportional hazards and Fine-Gray regression model, the early-onset LN group had a significantly better mortality rate than the late-onset LN group (*p* = 0.038 and *p* = 0.043, respectively).

**Conclusions:**

In our cohort, early-onset LN was a better predictor of CR at 6 and 12 months than late-onset LN. Our results suggest that early-onset LN patients had lower mortality than late-onset LN patients.

## Introduction

Systemic lupus erythematosus (SLE) is a chronic multisystem autoimmune disease with a wide range of clinical and immunological manifestations, among which lupus nephritis (LN) is the most common cause of morbidity and mortality [1]. In fact, between 50 and 60% of adult SLE patients develop signs and symptoms of kidney disease during their disease course [2, 3]. The standardized mortality rate of individuals with SLE without LN is 2.4-fold higher than that for the general population, while the rate for SLE patients with LN is much greater at 6.0–6.8-fold higher than that for the general population [4–7]. Although the mortality rate for SLE has declined over the past few decades, up to 20% of SLE patients who are still affected by LN will develop end-stage kidney disease (ESKD) within the first 10 years of the disease course [8, 9].

Most SLE patients who develop LN do so within 5 years of their diagnosis of SLE, but it is not uncommon for SLE patients to develop LN later than that [2]. It has been unclear whether the timing of the onset of LN influences the treatment response and long-term prognosis of the patients. Only a few studies have compared early-onset LN (occurring < 5 years after the diagnosis of SLE) with late-onset LN (occurring > 5 years post-diagnosis) [10, 11]. A recent report showed no difference in severity or long-term prognosis between early- and late-onset LN patients [11]. African and Hispanic patients are known to have worse renal outcomes and mortality than Caucasian patients, and there are differences in prognosis among ethnicities. To the best of our knowledge, no study has compared early-onset and late-onset LN using the above-described definition in an Asian population.

The recommendations for LN management published by the European League Against Rheumatism (EULAR)/European Renal Association-European Dialysis and Transplant Association (ERA-EDTA) aim for a complete renal response (CR) by 12 months of treatment, but this goal can be extended if nephrotic-range proteinuria is present at baseline [12]. We demonstrated that the survival rate of LN patients was significantly correlated with their attainment of a CR at 12 months after the start of induction therapy [13]. Many poor-prognosis factors for LN have been reported, including age, sex, ethnicity, and histological findings, and we speculated that the prediction of CR attainment at 6 and 12 months of treatment would lead to better renal outcomes and life prognoses [6, 14].

In this study, we determined the predictors of CR attainment after 6 and 12 months of induction therapy, and we examined the relationship between renal outcomes and mortality in early- and late-onset LN patients with biopsy-proven LN treated at Nagasaki University Hospital and affiliated community hospitals in Nagasaki, Japan.

### Patients and methods

This was a retrospective observational study comprising a total of 201 patients with biopsy-proven LN treated between 1990 and 2016 at Nagasaki University Hospital and affiliated community hospitals. The diagnosis of SLE in all patients was made by the attending physician according to the 1997 American College of Rheumatology (ACR) criteria [15]. Two expert nephropathologists (M.K. and T.T.) classified the biopsy specimens separately based on the International Society of Nephrology/Renal Pathology (ISN/RPS) classification to obtain the LN patients’ pathological information [16], regardless of the patients’ previous World Health Organization (WHO) or ISN/RPS classification. The ISN/RPS class II, III/IV, or V types were pure types and did not include any other types.

Patients with advanced comorbidities or other diseases associated with impaired renal function (e.g., diabetic or primary renal disease) were excluded. Patients with inadequate medical records and patients with < 12 months of follow-up were also excluded. All patients were followed up at 1- to 3-month intervals and at ≥ 12 months from the date of their renal biopsy.

We divided the 201 patients into two groups: early-onset LN and late-onset LN. As in previous studies [10, 11, 17], early onset was defined as the development of LN within 5 years of the patient’s SLE diagnosis, and late onset was defined as the development of LN > 5 years after the patient’s SLE diagnosis. Some of the patients provided written informed consent for the use of their data, and the opt-out strategy was used by the remainder of the patients. Patients who declined to give informed consent were excluded. The study was reviewed and approved by the Medical Ethics Committee of Nagasaki University Hospital (approval nos. 12012397 and 17082129).

### Data collection

The patients’ baseline characteristics were collected on the dates of their renal biopsies. The demographic data included the patient’s age at the onset of SLE, sex, duration of SLE (from the diagnosis of SLE to renal biopsy), and comorbidities of Sjögren’s syndrome (SS)/anti-phospholipid syndrome (APS) and the induction treatment used. We analyzed the patients’ laboratory data, including the white blood cell (WBC) count, lymphocyte count, hemoglobin, platelet counts, albumin, proteinuria, urine protein/creatinine ratio (Up/Ucr), serum creatinine (Cr), blood urea nitrogen (BUN), and estimated glomerular filtration rate (GFR). Immunological parameters were also measured, including complement 3 (C3), complement 4 (C4), total hemolytic complement (CH50), immunoglobulin (Ig) G, IgA, IgM, anti-nuclear antibody (ANA), anti-double-stranded DNA antibody (anti-dsDNA), anti-Smith (Sm) antibody, and anti-ribonucleoprotein (RNP) antibody. The histological characteristics of the activity and chronicity scores were determined as described previously [18]. We examined the presence or absence of hypertension at the time of renal biopsy. Hypertension was defined as systolic blood pressure ≥ 140 or diastolic blood pressure ≥ 90 mmHg on 2 or more occasions.

### Treatment and the definition of complete renal remission

Based on the clinical judgment of the rheumatologist and the treatment guidelines/recommendations for LN published by the ACR and the EULAR/ERA-EDTA [19, 20], the patient was treated with immunosuppressive agents. Treatment consisted of prednisolone (PSL) with intravenous cyclophosphamide (IVCY; 500–1000 mg/m^2^ body surface area 1×/month for 6 months), or PSL in combination with a first-line immunosuppressive regimen used for the treatment of LN, followed by IVCY or oral immunosuppressive agents quarterly; PSL was administered at doses of 0.5–1 mg/kg/day with or without intravenous methylprednisolone (mPSL) pulse therapy (50–1000 mg/day × 3 days). Plasma exchange (PE) was performed in patients who were refractory to other treatments.

At the discretion of the attending physician, induction therapy was performed for approx. 6 months. We defined CR at 6 and 12 months as an Up/Ucr ratio < 50 mg/mmol (roughly equivalent to proteinuria < 0.5 g/24 h) and a normal or near-normal GFR (within 10% of the patient’s normal GFR if previously abnormal). We defined partial renal response (PR) as a normal or near-normal GFR with a ≥ 50% reduction in proteinuria to subnephrotic levels [19, 21, 22].

### Mortality, the occurrence of ESKD, and predictors of CR attainment at 6 and 12 months

The primary outcome was mortality from any cause in both the early- and late-onset LN groups. The secondary outcome was ESKD, defined as dialysis dependence for > 3 months. We also determined the predictors of CR attainment at 6 and 12 months after the start of induction therapy. Data were collected until either the patient’s last follow-up or until December 31, 2019, whichever was later.

### Statistical analyses

A nonparametric Wilcoxon rank-sum test was used for intergroup comparisons of multiple variables. Fisher’s exact test was used to test for possible associations between each variable and the treatment response. We conducted univariate and multivariate regression analysis to determine the predictive factors of clinical response. The above statistical analyses were performed using JMP® Pro15 software (SAS Institute, Cary, NC). Data related to the length of time to ESKD or mortality after induction therapy were analyzed using the 1-*KM* (Kaplan-Meier estimate) method with a log-rank test. Patients were censored if they were lost to follow-up or reached the end of the study. Kaplan-Meier analyses may overestimate the cumulative incidence if death is censored in the same way as when censoring for other reasons; therefore, the cumulative incidence of ESKD and mortality were also analyzed with death as a competing risk by Gray’s test. A Cox proportional hazards and Fine-Gray regression model were used to examine the risk of ESKD and mortality. These calculations were performed with EZR (Saitama Medical Center, Jichi Medical University, Saitama, Japan), which is a graphical user interface for R (The R Foundation for Statistical Computing, Vienna, Austria) [23]. The significance level was set at *p* < 0.05.

## Results

### Patient characteristics

**Fig. 1:**
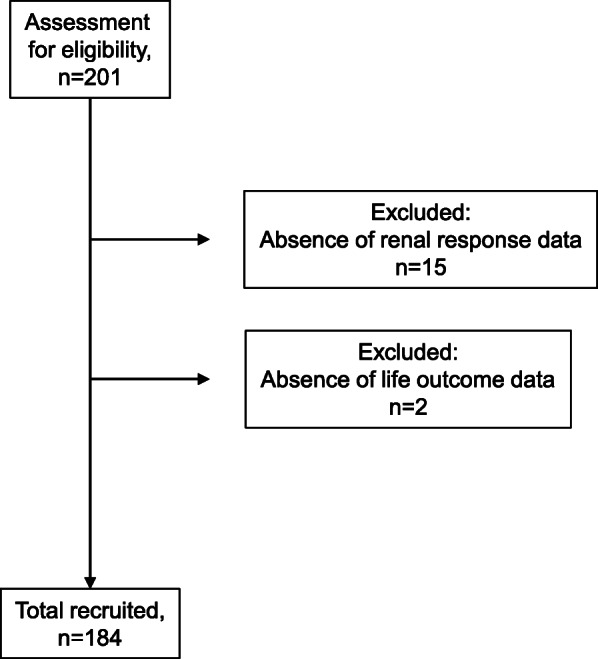
Patient enrollment flow: 201 patients with lupus nephritis (LN) were enrolled

Of the 201 enrolled patients, a total of 184 patients could be followed for therapeutic response at 6 and 12 months after their induction therapy (Fig. 1). The demographic and disease-related features of the 184 patients are summarized in Supplementary Table S1. The majority of the patients were female (84.8%). The median age at the onset of LN was 34.0 years (interquartile range [IQR] 24.0–45.0 years), and the disease duration of SLE was 21 months (IQR 1.0–116.0 months). The mean follow-up duration after the renal biopsy was 123 ± 85 months. The renal pathology of 99 (53.8%) patients was classified as ISN/RPS class III or IV, and 41 (22.2%) patients were classified as ISN/RPS class V. Seventy-seven (43.0%) patients were treated with intravenous mPSL pulse therapy, 41 (22.9%) patients were treated with IVCY, and 58 (32.4%) patients were treated with tacrolimus (TAC) for induction therapy.

We then divided the 184 patients into two groups based on whether they had early-onset or late-onset LN (Table 1): 113 patients (61.4%) had early-onset LN, and 71 patients (38.6%) had late-onset LN. Among the disease-related features at baseline, higher age at SLE onset (*p* < 0.001), a lower WBC count (*p* = 0.004), a higher ANA titer (*p* < 0.001), an elevated anti-ds-DNA antibody titer (*p* < 0.001), higher IgG and IgM levels (p < 0.001), lower CH50 (*p* = 0.016) and C3 (*p* = 0.001) levels, a lower prevalence of ISN/RPS class III or IV (*p* = 0.048), and a lower index of chronicity (0–12) (*p* < 0.001) were significantly related to early-onset LN.

**Table 1:** Baseline characteristics of the patients

Baseline variables	Early-onset LN (*N* = 113)		Late-onset LN (*N* = 71)		*p* value	Baseline variables	Early-onset LN (*N* = 113)		Late-onset LN (*N* = 71)		*p* value
	Median	IQR	Median	IQR			Median	IQR	Median	IQR	
Age at SLE onset, years	32	(21–45)	24	(16–31)	< 0.001*	CH50 (mg/dl)	16.8	(10.4–30.0)	18.9	(14.5–31.2)	0.016*
Age at LN onset, years	32	(22–46)	35	(29–44)	0.065	C3 (mg/dl)	41.2	(28.9–63.4)	56.5	(40.9–72.5)	0.001*
Sex (% female)	92/113(81.4)		64/71(90.1)		0.141	C4 (mg/dl)	8.0	(4.3–13.1)	10.0	(6.1–16.4)	0.069
SLE duration, months	2	(0–10)	125	(101–197)	< 0.001*	Comorbidities of SS (%)	15/113 (13.3)		7/71 (9.9)		0.642
Proteinuria, g/gCr	1.4	(0.6–3.8)	2.1	(1.0–3.4)	0.199	Comorbidities of APS (%)	13/113 (11.5)		5/71 (7.0)		0.446
White blood cell count, /μl	4700	(3720–6800)	5800	(4560–7725)	0.004*	ISN/RPS III or IV (%)	54/113 (47.8)		45/71 (63.4)		0.048*
Lymphocyte count, /μl	880	(554–1491)	918	(617–1400)	0.998	ISN/RPS V (%)	24/113 (21.2)		17/71 (23.9)		0.717
Hemoglobin, g/dl	11.0	(9.8–12.2)	11.5	(10.4–13.1)	0.066	Index of activity (0–24)	5	(3–7)	6	(4–8)	0.056
Platelet counts, ×10^4^/μl	20.7	(14.0–26.1)	22.2	(17.0–27.7)	0.174	Index of chronicity (0–12)	2	(0–2)	3	(2–4)	< 0.001*
Albumin, g/dl	3.2	(2.5–3.8)	3.2	(2.8–3.8)	0.324	mPSL pulse (%)	60/109 (55.1)		42/70 (60.0)		0.539
BUN, mg/dl	14.2	(11.0–20.0)	15.5	(12.0–21.3)	0.504	TAC (%)	30/109 (27.5)		28/70 (40.0)		0.102
Cr, mg/dl	0.7	(0.6–1.0)	0.7	(0.6–0.9)	0.906	CyA (%)	11/109 (10.1)		9/70 (12.9)		0.630
eGFR, ml/min/1.73 m^2^	80.6	(58.0–102.6)	78.8	(57.7–97.6)	0.560	AZP (%)	2/109 (1.8)		2/70 (2.9)		0.645
ANA	640	(175–1280)	320	(80–640)	< 0.001*	MZR (%)	28/109 (25.7)		18/70 (25.7)		1.000
Anti-ds-DNA antibodies, U/ml	50.8	(12.1–300.0)	20.8	(5.1–71.7)	< 0.001*	IVCY (%)	24/109 (22.0)		17/70 (24.3)		0.720
Anti-RNP antibodies, U/ml	8.7	(4.2–98.5)	8.8	(2.4–86.2)	0.403	MMF (%)	4/109 (3.7)		5/70 (7.1)		0.316
Anti-Sm antibodies, U/ml	8.7	(2.3–88.2)	4.4	(1.0–28.9)	0.095	PE (%)	9/109 (8.3)		5/70 (7.1)		1.000
IgG, mg/dl	1750	(1327–2190)	1150	(801–1482)	< 0.001*	Hypertension (%)	43/110 (39.1)		27/68 (39.7)		1.000
IgA, mg/dl	271	(196–369)	274	(188–369)	0.371	Biopsy before 2002	52/113 (46.0)		28/71 (39.4)		0.446
IgM, mg/dl	114.0	(75.2–175.0)	27.1	(45.8–142.3)	< 0.001*						

*p < 0.05. *p* values were determined by nonparametric Wilcoxon rank-sum test and Fisher’s exact test. *IQR i*nterquartile range

### Differences in the ISN/RPS classification between early-onset and late-onset LN

**Fig. 2:**
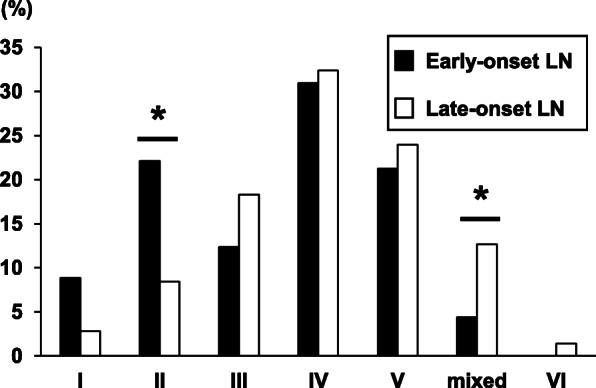
The differences in ISN/RPS classifications between the early- and late-onset LN groups. *p < 0.05

There were significantly more patients with pure class II nephritis in the early-onset LN group (*p* < 0.05) (Fig. 2), whereas class III or IV (III/IV) (Table 1) and mixed classes III + V and IV + V LN were significantly more prevalent in the late-onset LN group (*p* < 0.05) (Fig. 2). The prevalences of pure classes I, III, IV, V, and VI were not significantly different between the early- and late-onset groups.

### Predictors of CR at 6 months and 12 months after induction therapy

The predictors of a CR at 6 and 12 months after induction therapy in the univariate and multivariate analyses are shown in Tables 2 and 3, respectively. In the multivariate regression analysis, the independent predictors of a CR at 6 months after induction therapy were female sex (odds ratio [OR] 3.93, 95% confidence interval [CI] 1.31–11.77, *p* = 0.010), proteinuria (g/gCr) (OR 0.83, 95% CI 0.71–0.97, *p* = 0.009), index of activity (0–24) (OR 0.83, 95% CI 0.70–0.99, *p* = 0.030), and early-onset LN (OR 2.39, 95% CI 1.15–4.98, *p* = 0.018). The independent predictors of a CR at 12 months after induction therapy were female sex (OR 3.60, 95% CI 1.32–9.83, *p* = 0.013), mixed LN (OR 0.18, 95% CI 0.04–0.80, *p* = 0.024), index of activity (0–24) (OR 0.80, 95% CI 0.68–0.94, *p* = 0.007), and early-onset LN (OR 2.10, 95% CI 1.05–4.23, *p* = 0.035).

**Table 2:** Multivariate regression model of factors predictive of achieving a complete renal response at 6 months

	Univariate			Multivariate		
Parameter	OR	95% CI	***p*** value	OR	95% CI	***p*** value
Sex (% female)	3.67	1.41–9.54	0.004*	3.93	1.31–11.77	0.010*
ISN/RPS III or IV, %	0.40	0.22–0.73	0.002*	1.43	0.58–3.51	0.439
Mixed LN	0.64	0.21–1.99	0.433	–	–	–
Cr, mg/dl	0.42	0.19–0.91	0.011*	0.93	0.45–1.90	0.838
Proteinuria, g/gCr	0.77	0.66–0.90	< 0.001*	0.83	0.71–0.97	0.009*
Index of activity (0–24)	0.79	0.70–0.89	< 0.001*	0.83	0.70–0.99	0.030*
Hypertension	0.31	0.16–0.59	< 0.001*	0.65	0.30–1.41	0.279
Early-onset LN	2.01	1.09–3.70	0.024*	2.39	1.15–4.98	0.018*

*p < 0.05

**Table 3:** Multivariate regression model of factors predictive of achieving a complete renal response at 12 months

	Univariate			Multivariate		
Parameter	OR	95% CI	***p*** value	OR	95% CI	***p*** value
Sex (% female)	2.88	1.25–6.66	0.011*	3.60	1.32–9.83	0.013*
ISN/RPS III or IV, %	0.53	0.29–0.96	0.035*	1.82	0.73–4.50	0.192
Mixed LN	0.27	0.08–0.88	0.022*	0.18	0.04–0.80	0.024*
Hemoglobin, g/dl	1.16	0.99–1.35	0.066	1.18	0.97–1.43	0.086
Cr, mg/dl	0.52	0.27–0.99	0.031*	0.96	0.48–1.89	0.900
Index of activity (0–24)	0.81	0.73–0.91	< 0.001*	0.80	0.68–0.94	0.007*
Hypertension	0.44	0.24–0.81	0.009*	0.59	0.28–1.22	0.153
Early-onset LN	1.91	1.05–3.49	0.035*	2.10	1.05–4.23	0.035*

*p < 0.05

### The renal survival rate and survival rate: early-onset vs. late-onset LN

**Fig. 3:**
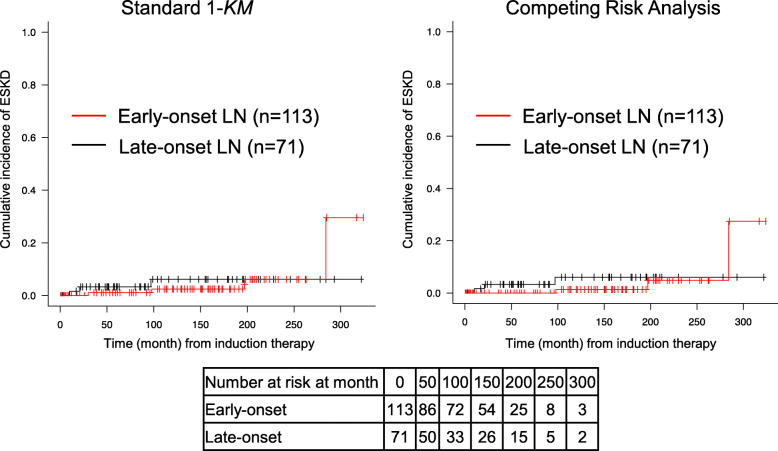
The 1-*KM* (Kaplan-Meier analysis estimate) and competing risk analysis of the cumulative end-stage kidney disease (ESKD) rate according to the early- and late-onset LN. Red line: the number of early-onset LN patients at each time point. Black line*:* the number of late-onset LN patients at each time point. The raw numbers of patients analyzed in each subset at each time point are included below the figures; these were patients whose ESKD was considered to be “at risk”

**Fig. 4:**
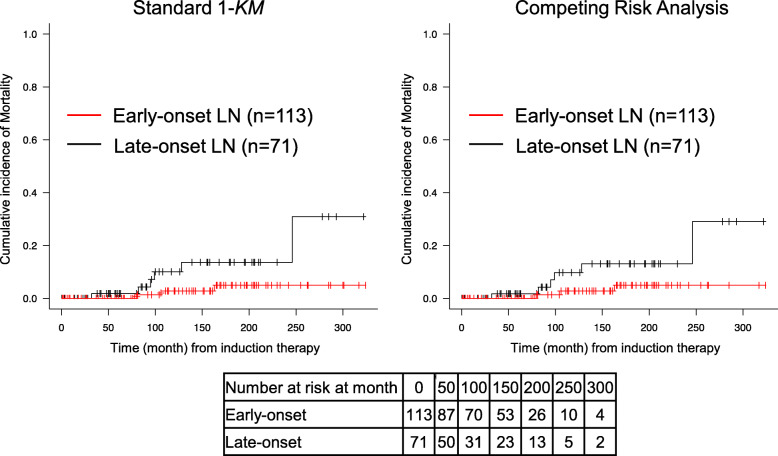
The 1-*KM* (Kaplan-Meier analysis estimate) and competing risk of the cumulative mortality rate according to the early- and late-onset LN. Red line: the number of early-onset LN patients at each time point. Black line: the number of late-onset LN patients at each time point. The raw numbers of patients analyzed in each subset at each time point are included below the figures; these were patients whose mortality was considered to be “at risk”

Seven patients (3.8%) progressed to ESKD, and nine patients (4.9%) died during the observation period. The 1-*KM* (Kaplan-Meier analysis estimate) and competing risk analysis showed that the cumulative incidence of ESKD was not significantly different between the early-onset LN group and late-onset LN group (*p* = 0.725 and *p* = 0.575, respectively) (Fig. 3), whereas the cumulative incidence of mortality differed significantly between the early- and late-onset LN groups (*p* = 0.031 and *p* = 0.040, respectively) (Fig. 4).

### The hazard risk of ESKD and mortality

In both the Cox and Fine-Gray regression models, the hazard ratio (HR) for ESKD in the early-onset LN group was not significantly different from that in the late-onset group. In contrast, the HR for mortality in the early-onset group was significantly lower than that in the late-onset group (Table 4). The result did not change when death was included in the cumulative incidence or Fine-Gray regression analysis as competing risk.

**Table 4:** Cox proportional hazards and Fine-Gray regression model for risk of end-stage kidney disease and mortality

	Cox regression model			Fine-Gray regression model		
Variables	End-stage kidney disease					
	Hazard ratio	95% CI	*p* value	Hazard ratio	95% CI	*p* value
Late-onset LN	1	Ref.		1	Ref.	
Early-onset LN	0.76	0.17–3.90	0.726	0.61	0.12–3.21	0.560
Variables	Mortality					
	Hazard ratio	95% CI	*p* value	Hazard ratio	95% CI	*p* value
Late-onset LN	1	Ref.		1	Ref.	
Early-onset LN	0.24	0.05–0.93	0.038*	0.26	0.07–0.99	0.043*

*p < 0.05

## Discussion

The results of our analyses demonstrated that early-onset LN was a predictor of CR attainment at 6 and 12 months of treatment. Several studies have indicated that patients with LN who attain a CR have a better survival rate than those who do not attain a CR [13, 24, 25]. We thus speculated that patients with early-onset LN would have a better mortality rate than those with late-onset LN.

Several studies compared the clinical characteristics of early-onset versus late-onset LN and their association with long-term prognosis. The studies’ conclusions varied and are controversial; Varela et al. compared early-onset and late-onset LN and reported no significant difference in nephritis development or histological type [10], and Ugolini-Lopes et al. observed no differences in serum Cr levels or the prevalence of ESKD or mortality after 7 years of follow-up [11]. A recent investigation comparing the disease profiles and outcomes of early-onset and late-onset LN patients did not reveal any significant differences [17]. However, these reports lack treatment information and clinicopathological considerations (including the index of activity and chronicity), which are study limitations.

In Japan, a study comparing early- and late-onset LN in a cohort other than ours was reported [26]; its authors defined early-onset LN as the development of LN within 1 year of the onset of SLE. They reported that their early-onset LN patients achieved a better response to treatment than their late-onset patients, which is similar to our present findings.

Most cases of LN develop within 5 years of the diagnosis of SLE, with approx. 5–15% of LN cases developing later [2, 27, 28]. There are no standardized definitions of early-onset and late-onset LN, but several studies made clinical comparisons separated by 5 years [10, 11, 17], and we followed that approach in the present work.

In our cohort, the predictors of CR after 6 and 12 months of induction therapy were female sex, proteinuria (CR attainment at 6 months only), mixed LN (CR attainment at 12 months only), index of activity (0–24), and early-onset LN. A number of LN studies have also reported that sex [29–31], proteinuria at baseline [13, 32], mixed LN [33], and index of activity (0–24) [34–36] affect the CR after induction therapy. However, to date, early-onset LN has not been reported to be an independent predictor of CR at 6 and 12 months.

In our cohort, the early-onset LN group was characterized by higher levels of anti-ds-DNA antibodies and hypocomplementemia with higher serological activity (Table 1), more frequent ISN/RPS class II (Fig. 2), less frequent class III/IV (Table 1) and mixed LN (Fig. 2), and a lower index of chronicity (0–12) compared to the late-onset group. We consider the possibility that the late-onset LN cases may not have been highly immunologically active compared to the early-onset LN cases, since treatment with some therapeutic agents had already started at baseline. More importantly, we suspect that this difference in attainment of a CR was observed because our early-onset LN group had more patients in ISN/RPS class II, and patients in this class are known to achieve a better response to induction therapy compared to those with class III/IV or mixed LN. Some investigators have proposed that long-term renal function was significantly affected by the baseline index of chronicity (0–12) in LN patients [37, 38].

Our present findings also showed that early-onset LN has a lower hazard ratio for mortality compared to late-onset LN (Table 4). In an earlier study, we observed that the survival rate of LN patients was significantly correlated with CR attainment at 12 months after the start of induction therapy [13]. We believe that early-onset LN has a higher CR attainment rate at 12 months compared to late-onset LN and that this may be associated with better mortality. The nine deaths in our cohort were due mainly to cardiovascular complications, malignancies, and infections (Suppl. Table S2). In our cohort, the duration of SLE at baseline was significantly longer in the late-onset LN group compared to the early-onset group, and the patients with late-onset LN may have had longer exposures to prednisolone and immunosuppressants. The patients’ compromised conditions, vascular lesions, and other complications from long-term prednisolone and immunosuppressant treatment may have affected their mortality. However, we were not able to collect information on the patients’ treatment before the diagnosis of LN for the present analyses.

There are some limitations of our study that deserve mention and suggest caution regarding the interpretation of the results. First, we must consider that a limitation of this type of study is that patients may have had a period of undiagnosed SLE before the diagnosis was made. Second, a selection bias for patients with LN may exist in this cohort. Since some patients with LN may have refused biopsy, the exclusion of these cases would have introduced a bias. In addition, there may have been variation in the Up/Ucr criterion (0.5 g or 1 g), which is important in the decision to perform a renal biopsy, and here again patient selection bias would have occurred. Third, the difference in findings between the two nephropathologists was not insignificant, and since treatment decisions are made based on what the treating pathologist reads, this could clearly have had an impact on their results, even if the study pathologist did not directly influence the treatment. Fourth, our cohort had a long-term follow-up period, and there was a variation in the protocol for induction therapy; in particular, we were unable to enroll patients treated with hydroxychloroquine (HCQ) because HCQ for SLE patients was approved relatively recently in Japan (September 2015). The proportion of patients excluded because of HCQ use would have been greater in this study than in previous studies. Fifth, in late-onset LN, the duration of SLE is longer, and complications such as vascular damage and hypertension due to prednisolone and immunosuppressive treatment should be considered. However, we were not able to collect information on the patients’ treatment before the onset of their LN. Sixth, we may not be able to entirely eliminate the impact of heterogeneity between early- and late-onset LNs on the outcomes. The definitions of early- and late-onset LNs vary among cohorts, and differences in response to treatment by ethnicity and socioeconomic status have led to a lack of consensus. It is thus necessary to conduct a larger-scale, multicenter international collaborative study to test the findings described herein.

## Conclusions

We retrospectively analyzed the association between the mortality rate and the form of disease onset with a mean 10-year follow-up in patients with LN. Our analyses revealed that early-onset LN, female sex, and a lower index of activity (0–24) were the factors most predictive of CR attainment at both 6 and 12 months. Early-onset LN was associated with better mortality compared to late-onset LN. We need to further investigate the factors that worsen treatment response and mortality in patients with late-onset LN.

## Supplementary information

**Additional file 1: Figure S1.** The attainment of a complete renal response (CR) after 6 and 12 months of induction therapy in the early- and late-onset LN groups. *p < 0.05. (PPTX 50 kb)

**Additional file 2: Table S1.** Baseline characteristics of the patients. **Table S2.** Causes of death.

## Data Availability

The collected data processed in this study are stored at the Departments of Immunology and Rheumatology, Nagasaki University Graduate School of Biomedical Sciences, Nagasaki, Japan.

## Abbreviations

ACR: American College of Rheumatology
Anti-dsDNA: Anti-double-stranded DNA
Anti-RNP: Anti-ribonucleoprotein
Anti-Sm: Anti-Smith
APS: Anti-phospholipid syndrome
AZA: Azathioprine
CR: Complete renal response
CyA: Cyclosporine
ERA-EDTA: European Renal Association-European Dialysis and Transplant Association
EULAR: European League Against Rheumatism
ISN/RPS: International Society of Nephrology/Renal Pathology Society
IVCY: Intravenous cyclophosphamide
LN: Lupus nephritis
MMF: Mycophenolate mofetil
MZR: Mizoribine
PE: Plasma exchange
PR: Partial renal response
SLE: Systemic lupus erythematosus
SS: Sjögren syndrome
TAC: Tacrolimus
Up/Ucr: Urine protein/creatinine ratio
WHO: World Health Organization
