# Supplementary material for: Comparison of complete renal response and mortality in early- and late-onset lupus nephritis: a multicenter retrospective study of a Japanese cohort
Source: Arthritis Res Ther. 2020 Jul 22;22:175. doi: 10.1186/s13075-020-02271-3 (PMC7374914; doi:10.1186/s13075-020-02271-3)
Supplement: Supplementary file 1 — Additional file 1: Figure S1. The attainment of a complete renal response (CR) after 6 and 12 months of induction therapy in the early- and late-onset LN groups. *p < 0.05. (PPTX 50 kb) [file 13075_2020_2271_MOESM1_ESM.pptx]

## Slide 1
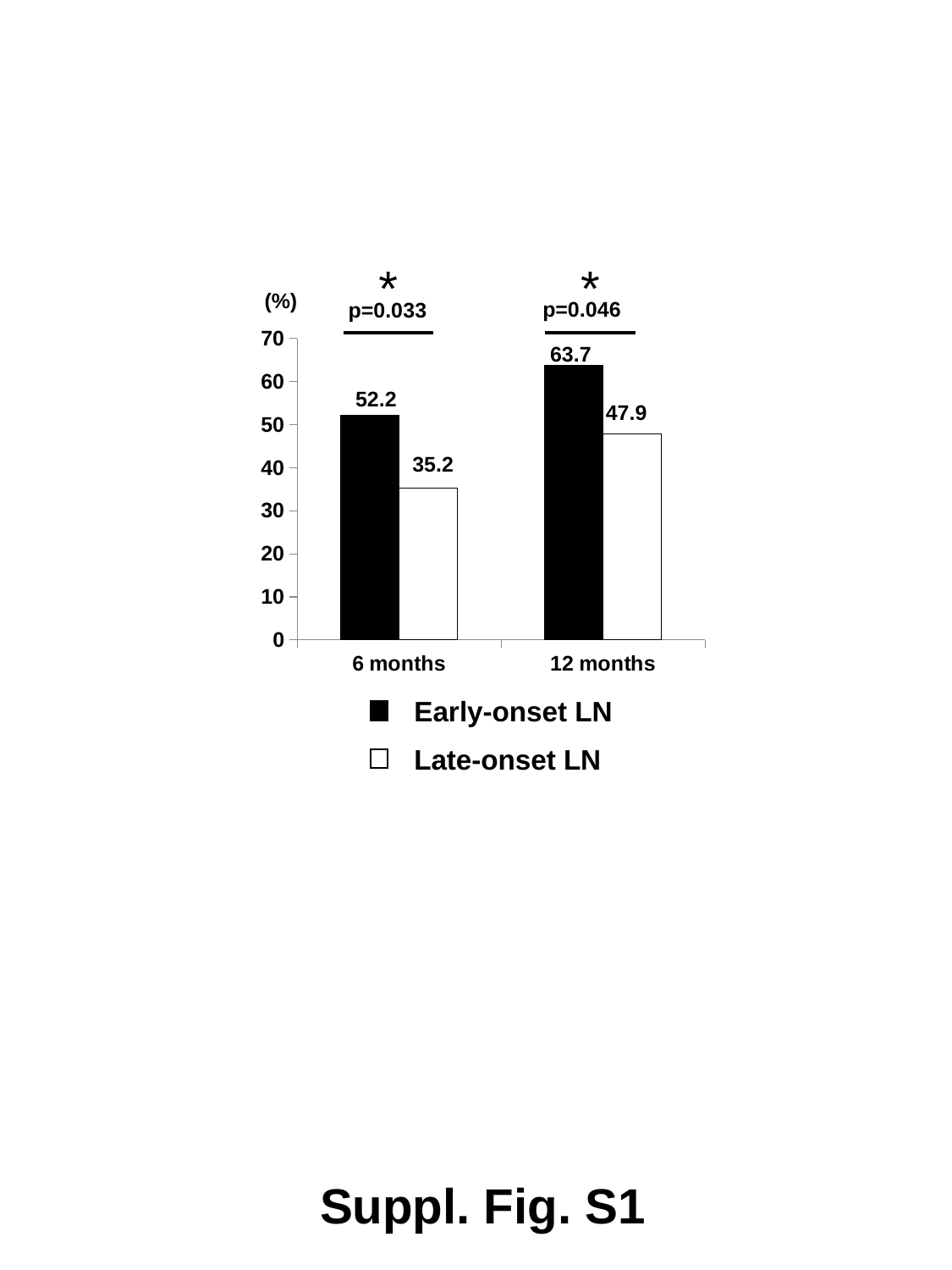

*
*
(%)
p=0.046
p=0.033
### Chart
| Category | early-onset LN | Late-onset LN |
|---|---|---|
| 6 months | 52.21 | 35.21 |
| 12 months | 63.72 | 47.89 |63.7
52.2
47.9
35.2
Early-onset LN
Late-onset LN
Suppl. Fig. S1
