# Supplementary material for: Comparison of complete renal response and mortality in early- and late-onset lupus nephritis: a multicenter retrospective study of a Japanese cohort
Source: Arthritis Res Ther. 2020 Jul 22;22:175. doi: 10.1186/s13075-020-02271-3 (PMC7374914; doi:10.1186/s13075-020-02271-3)
Supplement: Supplementary file 2 — Additional file 2: Table S1. Baseline characteristics of the patients. Table S2. Causes of death. [file 13075_2020_2271_MOESM2_ESM.docx]

**Supplementary Table S1.** Baseline characteristics of the patients

| Baseline variables | median | IQR | Baseline variables | median | IQR |
| --- | --- | --- | --- | --- | --- |
| Age at SLE onset, yrs | 27 | (19-39) | CH50(mg/dl) | 22.0 | (12.1-31.0) |
| Age at LN onset, yrs | 34 | (24-45) | Low CH50 (%) | 93/169 (55.0%) | |
| Sex (%female) | 156/184(84.8) | | C3(mg/dl) | 47.0 | (32.3-70.9) |
| SLE duration, months | 21 | (1-116) | Low C3 (%) | 154/180 (85.6%) | |
| Proteinuria, g/gCr | 1.6 | (0.7-3.6) | C4(mg/dl) | 8.5 | (5.0-14.5) |
| White blood cell count, /μl | 5100 | (3960-7225) | Low C4 (%) | 146/179 (81.6%) | |
| Lymphocyte count, /μl | 880 | (572-1432) | Comorbidities of SS (%) | 22/184(12.0) | |
| Hemoglobin, g/dl | 11.1 | (9.9-12.5) | Comorbidities of APS (%) | 18/184(9.8) | |
| Platelet counts, x10^4^/μl | 21.1 | (15.6-26.8) | ISN/RPS III or IV (%) | 99/184(53.8) | |
| Albumin, g/dl | 3.2 | (2.7-3.8) | ISN/RPS V (%) | 41/184(22.2) | |
| BUN, mg/dl | 15.0 | (11.8-21.0) | Index of activity(0-24) | 5 | (3-7) |
| Cr, mg/dl | 0.7 | (0.6-0.9) | Index of chronicity(0-12) | 2 | (1-3) |
| eGFR, ml/min/1.73 m^2^ | 79.1 | (58.0-100.4) | mPSL pulse(%) | 77/179(43.0) | |
| ANA | 640 | (160-1280) | TAC (%) | 58/179(32.4) | |
| Anti-ds-DNA antibodies, U/ml | 38.4 | (9.2-154.0) | CyA (%) | 20/179(11.1) | |
| Anti-ds-DNA antibodies, positive (%) | 126/180(70.0%) | | AZP (%) | 4/179(2.2) | |
| Anti-RNP antibodies, U/ml | 8.8 | (3.6-91.0) | MZR (%) | 46/179(26.0) | |
| Anti-Sm antibodies, U/ml | 6.5 | (1.8-45.8) | IVCY (%) | 41/179(22.9) | |
| IgG, mg/dl | 1494 | (1030-2038) | MMF (%) | 9/179(5.0) | |
| IgA, mg/dl | 271 | (195-390) | PE (%) | 14/179(7.8) | |
| IgM, mg/dl | 96.3 | (62.0-162.2) | Hypertension (%) | 70/178(39.3) | |
|  |  |  | Biopsy before 2002 (%) | 80/184(43.5) | |

**Suppl. Table S2.** Causes of death

| Cause of death | n |
| --- | --- |
| Cardiovascular disease | 3 |
| Infection | 1 |
| Malignancy | 1 |
| Others: |  |
| Pulmonary hemorrhage | 1 |
| Lupus enterocolitis | 1 |
| Unknown | 2 |
| Total | 9 |
